# Early-life milk replacer feeding mediates lipid metabolism disorders induced by colonic microbiota and bile acid profiles to reduce body weight in goat model

**DOI:** 10.1186/s40104-024-01072-x

**Published:** 2024-09-04

**Authors:** Ke Zhang, Ting Zhang, Mengmeng Guo, Awang Cuoji, Yangbin Xu, Yitong Zhao, Yuxin Yang, Daniel Brugger, Xiaolong Wang, Langda Suo, Yujiang Wu, Yulin Chen

**Affiliations:** 1 Key Laboratory of Animal Genetics, Breeding and Reproduction of Shaanxi Province, College of Animal Science and Technology, Northwest A&F University, Yangling, 712100 China; 2 College of Animal Engineering, Yangling Vocational and Technical College, Yangling , Shaanxi, 712100 China; 3 Institute of Animal Sciences, Tibet Academy of Agricultural and Animal Husbandry Sciences, Lhasa, 850009 China; 4 Institute of Animal Nutrition and Dietetics, Vetsuisse-Faculty, University of Zurich, Zurich, 8057 Switzerland; 5 Key Laboratory of Animal Genetics and Breeding On Tibetan Plateau, Ministry of Agriculture and Rural Affairs, Lhasa, 850009 China

**Keywords:** Bile acid, Colon microbiota, Goat model, Lipid metabolism, Milk replacer

## Abstract

**Background:**

Dysregulation of lipid metabolism and its consequences on growth performance in young ruminants have attracted attention, especially in the context of alternative feeding strategies. This study aims to elucidate the effects of milk replacer (MR) feeding on growth, lipid metabolism, colonic epithelial gene expression, colonic microbiota composition and systemic metabolism in goat kids compared to breast milk (BM) feeding, addressing a critical knowledge gap in early life nutrition.

**Methods:**

Ten female goat kids were divided into 2 groups: those fed breast milk (BM group) and those fed a milk replacer (MR group). Over a period of 28 d, body weight was monitored and blood and tissue samples were collected for biochemical, transcriptomic and metabolomic analyses. Profiling of the colonial microbiota was performed using 16S rRNA gene sequencing. Intestinal microbiota transplantation (IMT) experiments in gnotobiotic mice were performed to validate causality.

**Results:**

MR-fed pups exhibited reduced daily body-weight gain due to impaired lipid metabolism as evidenced by lower serum and liver total cholesterol (TC) and non-esterified fatty acid (NEFA) concentrations. Transcriptomic analysis of the colonic epithelium revealed upregulated genes involved in negative regulation of lipid metabolism, concomitant with microbiota shifts characterized by a decrease in Firmicutes and an increase in Actinobacteria. Specifically, genera such as *Bifidobacterium* and *Prevotella* were enriched in the MR group, while *Clostridium* and *Faecalibacterium* were depleted. Metabolomics analyses confirmed alterations in bile acid and fatty acid metabolic pathways. IMT experiments in mice recapitulated the metabolic phenotype observed in MR-fed goats, confirming the role of the microbiota in modulating host lipid metabolism.

**Conclusions:**

Milk replacer feeding in goat kids disrupts lipid metabolism and gut microbiota dynamics, resulting in reduced growth rates and metabolic alterations. These findings highlight the importance of early nutritional intervention on metabolic programming and suggest that modulation of the gut microbiota may be a target for improving growth and metabolic health in ruminants. This study contributes to the understanding of nutritional management strategies in livestock and their impact on animal health and productivity.

**Supplementary Information:**

The online version contains supplementary material available at 10.1186/s40104-024-01072-x.

## Background

In mammals, breastfeeding is universally recognized as the normative and superior nutritional foundation for newborns, primarily due to its synergistic combination of nutrients and bioactive compounds that confer significant biological effects [1]. The positive influence of breastfeeding on the health, growth and development of the offspring from a nutritional, physiological and developmental point of view is well established [2]. Breast milk supports the development of the immune system by providing age-appropriate nutrients, growth factors, antimicrobial peptides and proteins that meet the needs of the offspring [3]. However, in the context of intensive livestock production, particularly in goat production systems, the use of milk replacers (MR) is widespread due to limitations such as inadequate lactation, mastitis and postpartum paresis in ewes [4]. MR is used to compensate for the lack of maternal milk, improve litter survival and shorten the reproductive cycle of ewes [5]. Although current milk replacers are formulated to mimic the nutritional profile of breast milk using non-milk protein substitutes, they are notably lacking in many of the biologically active compounds found in natural milk. This disparity highlights the urgent need for systematic research aimed at identifying and selecting functional microorganisms and metabolites that promote the growth and maturation of offspring. These targeted microbial strains and metabolites could potentially be incorporated into novel milk replacers enriched with bioactive compounds.

The complex interplay between early life nutrition, gut microbiota composition, bile acid metabolism and lipid homeostasis have profound implications for the lifelong health and weight management of ruminants such as goats and other animals [6]. During the neonatal period, ruminants undergo a number of critical physiological adaptations that are essential for survival and future productivity. The process of ingestion of maternal milk involves the establishment and development of a diverse gut microbiota that plays an essential role in digestion, absorption [7] and metabolism of nutrients, particularly lipids [8–10]. When the natural supply of goat milk is limited or unavailable, feeding milk replacers is a standard practice to ensure adequate nutrition for rapidly growing young ruminants [11]. However, the extent to which this artificial feeding strategy affects the establishment and functionality of the gastrointestinal microbiota, particularly in the colon where bacterial fermentation is pronounced, remains an under-explored area of research. Bile acids, synthesized in the liver and secreted into the small intestine, play a central role in lipid digestion and absorption, while also serving as key regulatory signals involved in various aspects of host metabolism, including glucose-lipid balance and inflammatory pathways [12, 13]. Perturbations in the gut microbiota can induce alterations in bile acid metabolism, often leading to perturbations in bile acid pools that are closely associated with lipid metabolism disorders in different species [14, 15]. In this study, we aimed to unravel the complex interrelationships by investigating how early life milk replacer feeding affects the colonic microbiota and bile acid profiles, thereby affecting lipid metabolism and body weight in goat kids. We hypothesize that currently available commercial goat milk replacer formulae may exacerbate lipid metabolic disorders in young goats, thereby affecting their growth performance.

To address these knowledge gaps, we used milk replacer-fed goat models to systematically evaluate the effects of formula feeding on growth, microbiota composition and metabolic performance. In addition, we validated the causality of microbial dysbiosis-mediated disturbances in lipid metabolism using a pseudo germ-free mouse model. The overall aim of this investigation is to provide a comprehensive understanding of the proposed mechanisms by which milk replacers affect gut metabolism and physiology. Our research aims to contribute to the development of innovative milk replacer products and to improve the scientific understanding of milk replacer feeding practices.

## Materials and methods

The experiment was approved by the Institutional Animal Care and Use Committee of the Northwest A&F University under permit number 2020-03-015.

### Animals, diets, and experimental design

**Fig. 1:**
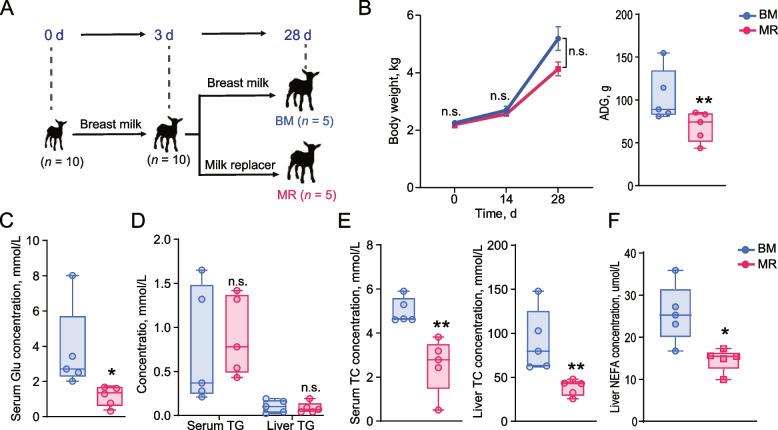
Effects of formula feeding on body weight and serum parameters in goats. **A** Schematic representation of the experimental design. **B** Graphical representation of changes in body weight and average daily gain of goats subjected to formula feeding. **C** Serum glucose concentration in goats under different feeding conditions. **D** Comparison of triglyceride (TG) concentrations in both serum and liver tissues of goats in different feeding groups. **E** Comparison of total cholesterol (TC) concentrations in both serum and liver tissues of goats in different feeding groups. **F** Non-esterified fatty acid (NEFA) concentrations in the liver of goats under different feeding conditions. Data differences among goat subjects were statistically assessed using one-way analysis of variance (ANOVA) with Tukey’s test. Statistical significance levels are denoted as follows: ns (not significant) for *P* > 0.05, * for *P* < 0.05, and ** for *P* < 0.01

Prior to the start of this study, 50 female Tibetan cashmere goats were tested for estrus by allowing visual and olfactory contact with an intact male goat, and a total of 36 female goats were selected on the basis of number of litters (2 times). These 36 goats (aged 24 months; mean live weight 25.58 kg) were reared and maintained under the same conditions at the Lhasa Tibet Cashmere Goat Breeding Farm (Lhasa, China). The selected goats had no history of diarrhea or other digestive disorders before and during the study. In addition, they were not given any medications, including antibiotics and probiotics, during the study. The diets and nutrient composition fed to the goats during the study are shown in Additional file 1: Table S1. The goats had free access to water and feed and the diet was not changed throughout the study. Following the pre-evaluation, 30 female goats were selected for treatment to induce concurrent estrus and subsequent mating, based on our standard pre-production procedure [16], due to the irregular estrus cycles observed in the other 20 female goats. Ultimately, 18 goats were successfully conceived and delivered, and a total of 10 singleton female goat kids of similar weight (mean weight 2.23 kg) were selected for subsequent experimental treatment. Ten female kids were fed breast milk ad libitum for 3 d after parturition, and on the 4^th^ d after birth, the kids were randomly divided into 2 groups (*n* = 5), with no difference in initial mean body weight between the two groups. To ensure consistency in the test environment, bulk milk samples from goats were used to make up the milk fed to the goat kids. The milk for the animals in the BM group was collected daily from the corresponding mothers of the kids. These mothers were milked daily to obtain the required bulk milk samples. Kids in the BM group were fed 4 times a day (at 8:00, 12:00, 18:00, and 22:00) with 250 mL of milk each time. Goat kids in the MR group were fed milk replacer 4 times a day (at 8:00, 12:00, 18:00 and 22:00) by mixing 40 g of commercial milk replacer (Beijing Precision Animal Nutrition Research Center, Beijing, China [8]) with 250 mL of warm water and bottle feeding each kid. This formulation meets or exceeds recommended levels for crude protein, crude fat, essential amino acids such as lysine, methionine, and threonine, as well as providing necessary vitamins and minerals including calcium, phosphorus, sodium chloride, and vitamin E. Each group of kids was housed in separate pens (6 m × 5 m; *n* = 5) in the same environment. All goat kids had free access to clean water and were offered fresh dried alfalfa ad libitum every day. The kids were already accustomed to consuming forage from 14 d. Observations of their ingestive behavior indicated that they consumed the alfalfa regularly, which was a standard part of their diet. This inclusion of dry alfalfa did not have any noticeable adverse effects on the results, as it ensured a consistent and natural feeding behavior among the kids. Goat’s milk at 7 d contained 9.52% protein, 7.50% fat, 14.95% solids-non-fat, 3.99% lactose, 2.58% low lactose, 1.29% galactose and 6.36% casein. Goat’s milk at 14 d contained 5.91% protein, 6.70% fat, 12.14% solids-non-fat, 4.16% lactose, 2.46% low lactose, 1.01% galactose and 4.31% casein. The formula of the milk replacer is shown in Additional file 1: Table S2. All kids were weighed at 0, 14, and 28 d after birth. At 29 days of age, after 12 h of fasting, 10 female goat kids from the experiment were sacrificed. Jugular vein blood was collected from the goats 1 h before slaughter, preserved in a vacuum tube containing coagulant, kept at 4 °C for 3 h, and then centrifuged at 2,000 × *g* at 4 °C for 10 min. They were euthanized by injection with thiopental (0.125 mg/kg body weight) and potassium chloride (5 to 10 mL) (Fig. 1A). The homogenized digesta from the colon was then snap frozen in liquid nitrogen and stored at −80 °C for subsequent DNA analysis. Colonic epithelial tissue was excised from the mucosal layers using a glass slide, immediately washed in ice-cold phosphate-buffered saline (PBS) until the PBS was clear, and then transferred directly to liquid nitrogen until tissue RNA extraction.

Five goat kids (mix pool of colon content of each group) from BM and MR groups were used for colonic digesta transplantation. The colonic content (200 mg) from the donors was suspended in sterile 0.9% saline (2 mL) and centrifuged at 1,000 × *g* at 4 °C for 5 min to obtain the bacterial suspension. Thirty-two female C57/6 J mice (8 weeks) were divided into 4 groups: Con, a control group (without any treatment); Ab, an antibiotic group (which received sterile saline via gavage after antibiotic treatment stopped); BM_IMT, a group that was transplanted with group BM colonic microbiota after antibiotic treatment stopped; and MR_IMT, a group that was transplanted with MR colonic microbiota after antibiotic treatment stopped. Ab, MR_IMT, and BM_IMT groups were first treated with fresh composite antibiotics (containing 1 g/L ampicillin, 1 g/L streptomycin, 1 g/L gentamicin, and 0.5 g/L vancomycin; MACKLIN, Shanghai China) using water for 14 d [17]. Each recipient mouse received 200 μL of the supernatant by oral gavage once a day continuously for 21 d. To improve the colonic microbial survival rates during the cryopreservation, colonic content samples were diluted with sterile saline, homogenized and filtered. The formula of the mice diet was shown in Additional file 1: Table S3. Subsequently, the resulting suspension was added to glycerol to get a final concentration of 10%. Finally, the colonic suspensions are labeled accurately, and stored in liquid nitrogen as soon as possible to ensure colonic microbial survival. When there is the need for IMT, the frozen colon suspension was thawed at 37 °C (water bath). Upon frozen colon suspension thawing, sterile saline solution can be added to obtain a required concentration (CFU = 1 × 10^9^) and the infusion of colon suspension should be implemented as soon as possible at 37 °C. All colonic material preparation processes were carried out at an anaerobic incubator (LAI-3 T, Longyue, China). After transplantation, they were housed individually at room temperature (23 ± 1 °C) and light control (16L:8D) with free access to feed and drinking water. Body mass was measured every day. Mice were sacrificed after 7 d of IMT (at 10:00), and the serum, colon contents, and colon tissue were collected after the mice were euthanized via CO_2_ inhalation then killed through cervical dislocation. All samples were stored at −80 °C for subsequent DNA analysis. Colonic tissue was stored in 4% paraformaldehyde in a refrigerator at 4 °C.

### Serum and hepatic parameters

The levels of serum total cholesterol (TC), triglycerides (TG), glucose (Glu), cortisol (Cor), corticosterone (Cort), diamine oxidase (DAO) and hypoxia-inducible factor (HIF-α) were measured using the HY-50061, HY-50062, HY-50063, HY-10062, HY-10063, HY-M0046 and HY-NE021 kits (Huaying, Beijing, China) on an automatic biochemical analyzer (Hitachi7160, Tokyo, Japan). Serum immunoglobulin A (IgA), immunoglobulin G (IgG), and immunoglobulin M (IgM) were measured by the sandwich ELISA method using the IgG/A/M (HY-50094) kit (Huaying, Beijing, China). Serum levels of interleukin-6 (IL-6), interleukin-10 (IL-10), tumor necrosis factor-α (TNF-α), and interleukin-1β (IL-1β) were measured using ELISA kit in accordance with the manufacturer’s instructions (Huaying, Beijing, China). Around 0.10 g of semi-thawed liver were homogenized an equivalent amount (1/9) of homogenizing buffer (pH 7.4; 0.01 mol/L). Screw tubes filled with 0.1 mmol/L EDTA-Na_2_, 0.8% NaCl, and Tris–HCl. The tubes were then put in frozen water for 5 min, shaken for 5 min at 25 Hz/s with a TissueLyser, and the procedure was performed once more. Then, using assay kits from Nanjing Jiancheng Bioengineering Institute (Nanjing, China), the supernatant was collected after centrifuging it for 10 min at 2,500 × *g* at 4 °C to test the levels of hepatic TC, TG, and non-esterified fatty acid (NEFA) (A111-101, A110-1-1, A042-1-1). The findings from the hepatic lipid test were adjusted using the total protein concentration in accordance with the manufacturer’s instructions (Nanjing Jiancheng Bioengineering Institute, Nanjing, China).

### 16S rRNA gene profiling

A total of 32 colonic content samples were collected from a mouse model after previous colonic digesta transplantation. Total DNA was then extracted from the cecum lumen content using the E.Z.N.A. stool DNA kit (Omega Bio-Tek, Norcross, GA, USA), according to the manufacturer’s instructions. The Nanodrop 2000 UV-VI spectrophotometer (Thermo Scientific, Wilmington, USA) was used to determine the DNA concentration and purity. 1% agarose gel electrophoresis was used to assess the quality of the extracted DNA. The primers 338F (5′-ACTCCTACGGGAGGCAG CAG-3′) and 806R (5′-GGACTACHVGGGTWTCTAAT-3′) on a thermocycler PCR system (Gene Amp 9700, ABI, USA) were used to amplify the V3–V4 region of the DNA [18]. The paired-end sequencing (2 × 300 bp) on an Illumina MiSeq platform (Illumina, San Diego, USA) was conducted according to the standard Major Biobio-Pharm Technology Co., Ltd. (Shanghai, China) protocols on the pooled equimolar ratios of the purified amplicons.

### Metagenomic sequencing, assembly and construction of the gene catalog

The DNA was extracted from colon content samples of goat using the E.Z.N.A Stool DNA kit (Omega Bio-tek, USA) according to the manufacturer’s instructions. The 1% agarose gel electrophoresis was used to evaluate the DNA quality and integrity, and DNA concentration was measured using the NanoDrop 2000 UV-VI spectrophotometers (Thermo Scientific, Wilmington, USA).

The DNA was fragmented by Covaris M220 (Genetics, China) to screen fragments of about 300 bp for subsequent construction of PE libraries. Metagenomic sequencing was conducted according to the standard protocols of Shanghai Majorbio Bio-pharm Technology Co., Ltd. (Shanghai, China). Sequence reads were treated to remove low-quality reads, trim the read sequences, and remove the host genome sequences. Specifically, reads with sequence lengths less than 50 bp and low-quality bases (quality value ≤ 20). Host genomic sequences were removed by Bowtie2 (v 2.2.9) software [19]. MEGAHIT (v 1.1.2) [20] were used to align the clean data of each sample to contigs (≥ 300 bp). MetaGene [21] (http://metagene.cb.k.u-tokyo.ac.jp/) was performed for predicting the open reading frames according to contigs (≥ 300 bp), and genes with nucleic acid lengths greater than 100 bp were selected and translated into amino acid sequences. Afterwards, CD-HIT (v 4.7) [22] was used to clustered (95% identity, 90% coverage), and the longest gene in each class was as the representative sequence to construct the non-redundant gene set. SOAPaligner (v 2.21) [23] was used to compare the high-quality reads obtained from the sequencing of single samples with the non-redundant gene set (95% identity), and to calculate the abundance data of each target gene in the corresponding samples.

After that, the constructed non-redundant (NR) gene was subjected to species and functional annotation, and the annotation process was conducted according to the standard protocols of Shanghai Majorbio Bio-pharm Technology Co., Ltd. (Shanghai, China) [24]. BLASTP (v 2.3.0) was used to blast the non-redundant gene set to the sequences of the NR database (e-value set to 1e-5) to obtain abundance data corresponding to each species. The non-redundant gene set sequences were aligned with KEGG database using BLASTP (v 2.3.0; e-value set to 1e-5). The annotation information of KEGG Ortholog (KO) from the KEGG database was acquired based on the relative abundance profile. HMMSCAN (v 3.1b2) [25] software was used to compare the amino acid sequence to the CAZyme (v 6.0) database (e-value set to 1e-5) and to obtain an annotation of the carbohydrate-active enzyme.

### RNA isolation and RNA-seq analyses

Total RNA was extracted from colon epithelial tissues of goat kids using TRIzol reagent (Invitrogen) according the manufacturer’s instruction. The RNA quality was evaluated using the Nanodrop 2000 (NanoDrop Technologies) and Bioanalyzer 2100 (Agilent). The Agilent 2100 was used to determine the RNA integrity number (RIN) value of the sample RNA. All RNA sample had the RIN value greater than 8.0 (RIN number: 8.20, 9.30, 8.40, 9.60, 9.40 in BM group sample, respectively, 9.40, 9.50, 9.40, 9.30, 8.60 in MR group sample, respectively). The RNA-seq transcriptome library was prepared using 5 µg of total RNA, following the TruSeq stranded RNA sample preparation kit for Illumina (San Diego, CA, USA). RNA-Seq library was sequenced with the Illumina NovaSeq 6000 platform (2 × 150 bp read length). The raw reads were trimmed and quality controlled by the SEQPREP and Sickle software. The TopHat (v 2.1.1) [26] software was used to align the clean data with the *Capra hircus* reference genome (GCA001704415.1) to obtain mapped data.

### Colonic content and serum metabolite measurements using LC–MS/MS

The colon content was homogenized by add ice-cold methanol/water (70%, v/v) in a ratio of 500 μL per 50 mg and then vortex for 3 min. The sample vortexed liquid was left at −20 °C for 30 min and centrifuge for 5 min at 4 °C and 12,000 × *g*. The collected supernatant was subjected to LC–MS/MS analysis (UPLC, Shim-pack UFLC SHIMADZU CBM A system; MS, QTRAP System). Serum samples were prepared by add 300 μL of methanol/water (70%, v/v) to 50 μL of serum, and vortexed for 3 min. The liquid was centrifuge for 10 min at 12,000 × *g* at 4 °C. The filtered samples were transferred to LC–MS/MS analysis.

### Quantitative PCR (qPCR) analysis

Total RNA was extracted using Trizol reagent (CWBIO, China) from colon tissues, and qPCR was performed using LightCycler 96 (Roche, NC, USA). qPCR primers are shown in Additional file 1: Table S4. All primers were obtained from Zhongke Biotechnology (Xi’an, China). The amplification efficiencies were 90%–110%. The reaction conditions were, 95 °C for 30 s, 95 °C for 10 s and 60 °C for 30 s for 40 cycles. Five biological replicates were set for each group. To minimize operational and technical errors, three technical replicates were performed for each sample. The expression of the target gene relative to the internal reference gene β-actin was calculated using the ΔΔCt method [27].

### Statistical analyses

GraphPad Prism (v9.0.0, GraphPad, USA) was used to generate histograms. The two-group data were subjected to independent sample *t*-test via SPSS (v 21.0, IBM, USA) to evaluate significance. The data are expressed as mean with standard error. Beta diversity analysis used the Bray-Curtis distance algorithm, and analysis of Similarities (ANOSIM) test was used to performed significant differences between groups with 999 permutations. The Kruskal-Wallis H test was used to identify significant differences of the relative abundance at different taxonomic levels between groups. Spearman correlation network analysis used python (v.2.7) stats, and the absolute value of the correlation coefficient was set to 0.8 and *P* ≤ 0.05. Linear discriminant analysis effect size (LEfSe) was performed to identified the microbiome with higher relative abundance in the two groups. The non-parametric factorial Kruskal-Wallis rank sum test was used, followed by linear discriminant analysis (LDA) to evaluate the impact of each taxon abundance on the differential effect. A significant increase in microbiota abundance was defined as an LDA score (log10) greater than 3.0. Metabolites with VIP ≥ 1 and *P* value < 0.05 were generally considered to be significantly different. R package (heatmaply; Complex Heatmap) was used to draw heatmaps of significant metabolites, and R (igraph) was used for correlation analysis in metabolite correlation network diagrams. DESeq2 software [28] based on negative binomial distribution was used to analyze raw counts, and genes with comparative expression differences between groups were obtained based on the default parameters *P*-adjust < 0.05 and |log_2_FC| ≥ 1 filtering conditions. Goatools was used to perform GO enrichment analysis on the genes in the gene set [29]. The method used was Fisher’s exact test, when the corrected *P*-adjust < 0.05.

## Results

### Dysregulation of lipid metabolism appears to be associated with the reduced daily weight gain observed in goat kids fed with milk replacer

Evaluating the effect of milk replacer on the growth performance of goat kids, we found that there was no significant difference in body weight between BM and MR groups on 0, 14 and 28 d after delivery, however, goats in the BM group gained weight significantly faster than those in the MR group, where kids in the BM group gained 104.64 g/d compared to 69.00 g/d in the MR group (*P* > 0.05; Fig. 1B). Compared with BM kids, stress-related indicators showed a highly significant increase in serum concentrations of Cor, Cort, HIF-α and DAO in the MR group (*P* < 0.001; Additional file 1: Fig. S1A), indicators related to pro-inflammation showed a highly significant increase in serum concentrations of IL-1β, IL-6 and TNF-α in the MR group, and a significant decrease in IL-10, an indicator related to anti-inflammation (*P* < 0.01; Additional file 1: Fig. S1B), consistent with immune-related indicators including IgA, IgG and IgM showed a similar pattern of decrease (*P* < 0.01; Additional file 1: Fig. S1B). The serum Glu index was also significantly decreased in the MR group (*P* < 0.05; Fig. 1C). To clearly clarify the effect of milk replacer feeding on lipid metabolism in kids, TC, TG and NEFA indictors were measured in serum and liver samples and revealed that milk replacer feeding treatment decreased TC and NEFA concentrations (*P* < 0.05; Fig. 1E and F), and no significant effect on TG concentrations in serum and liver (*P* > 0.05; Fig. 1D). Taken together, these results revealed that milk replacer feeding attenuates weight gain of kids and the lipid metabolism associated with it.

### Colonic epithelial lipid transport-related genes profile is influenced by milk replacer feeding

**Fig. 2:**
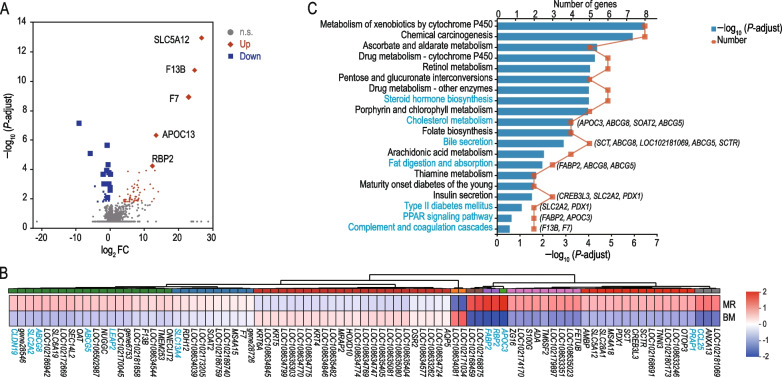
Transcriptional profiling of colonic epithelium in response to formula feeding. **A** Volcano plots illustrating the results of RNA-seq analyses comparing the colonic epithelium of goats in the BM (Breast Milk) and MR (Formula Feeding) groups. The red diamond indicates upregulated genes in the MR group, while the blue diamond represents downregulated genes in the MR group. **B** Heatmap displaying the differentially expressed genes (DEGs) in the MR and BM groups. **C** Kyoto Encyclopedia of Genes and Genomes (KEGG) enrichment analysis of DEGs in the BM vs. MR group. The genes associated with each pathway are listed in the respective bar

To further investigate the impact of milk replacer feeding on the expression profile of colonic epithelial lipid transport-related genes. We performed transcriptome sequencing using goat colonic epithelium samples. A total of 97.42 Gb clean data was obtained using RNA-seq of 10 colonic epithelium samples, with an average of 668,563,298 high-quality paired reads produced per sample. The alignment rate to the *Capra hircus* reference genome exceeded 94%. In total, 672 differentially expressed genes (DEGs) were screened, of which 403 were upregulated, and 269 were downregulated (Fig. 2A; Additional file 2: Tables S5). Among them, genes such as *ABCG8*, *ABCG5*, *SCTR*, *SCT*, *CCL25*, *PRAP1*, *FABP2*, *RBP2*, *APOC3*, *SLC5A12*, *CLDN19*, *SLC2A2*, *SLC13A4*, LOC102172669, LOC102181858, LOC102186942 and LOC102186759 were significantly upregulated in the MR group. In contrast, the genes *AQP5*, *OSR2*, *HOXD10*, *MRAP2*, *KRT4* and *KRT6A* were significantly downregulated in the MR group (|log_2_FC| ≥ 1 and *P*-adjust < 0.05; Fig. 2B). Among them, this DEGs mainly enriched in cholesterol metabolism pathway (involved in *APOC3*, *ABCG8*, *SOAT2* and *ABCG5*), steroid hormone biosynthesis pathway, bile secretion pathway (involved in *SCT*, *ABCG8*, LOC102181069, *ABCG5* and *SCTR*), fat digestion and absorption pathway (involved in *FABP2*, *ABCG8* and *ABCG5*), insulin secretion pathway (involved in *CREB3L3*, *SLC2A2* and *PDX1*) and PPAR signaling pathway (involved in *FABP2* and *APOC3*) (Fig. 2C). They were also enriched in 336 Gene Ontology (GO) categories, mainly including negative regulation of cholesterol transport, negative regulation of sterol transport, negative regulation of intestinal lipid absorption, negative regulation of intestinal cholesterol absorption, and negative regulation of intestinal phytosterol absorption (Additional file 1: Fig. S2A). Based on the functional enrichment analysis network, the significantly enriched pathways mainly involve downstream pathways related to negative regulation of cholesterol transport (Additional file 1: Fig. S2B). Taken together, milk replacer feeding significantly upregulates the expression of genes associated with negative regulation of lipid metabolism in the colon, leading to lipid dysfunction in goat kids.

### Milk replacer feeding altered the colonic microbiota and potential function in goat kids

**Fig. 3:**
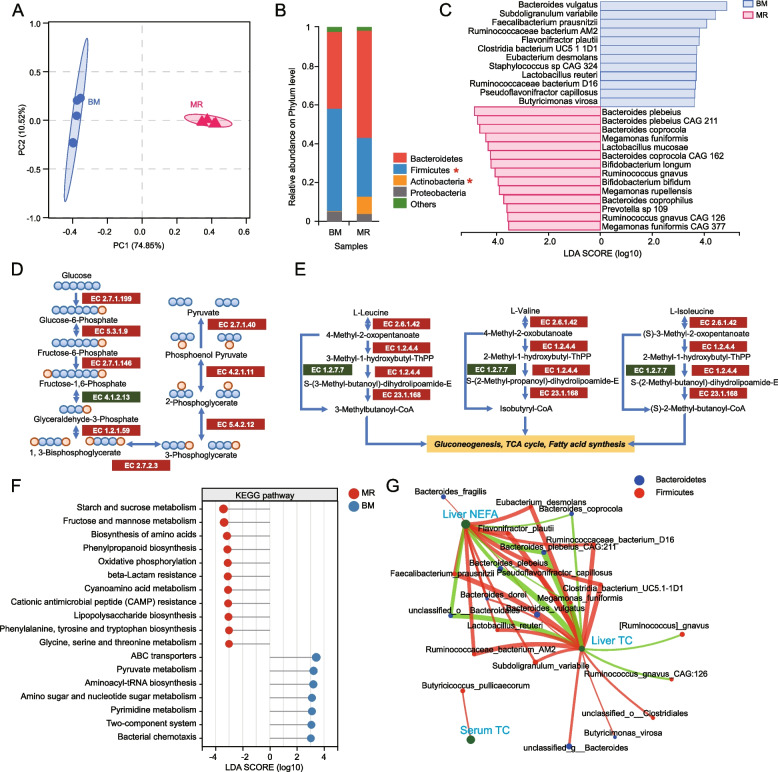
Effects of formula feeding on colonic microbiota and potential functional changes in goat kids. **A** Principal coordinate analysis (PCoA) plot based on relative species abundances, with box plots illustrating Bray–Curtis distances associated with groupings, assessed using the Wilcoxon rank-sum test. Analysis of similarity (ANOSIM) was employed to evaluate the dissimilarity of Bray–Curtis distances. **B** Relative abundance of bacterial phyla in the BM (Breast Milk) and MR (Formula Feeding) groups. Red asterisks indicate significant differences in bacterial abundance between the two groups. **C** Identification of bacterial species that exhibit differential abundance between groups, as determined by linear discriminant analysis effect size (LEfSe) analysis. The analysis employed a one-against-all multi-group comparison strategy, with a linear discriminant analysis (LDA) threshold set at > 3.0. **D **and** E** Variations in the abundance of KEGG (Kyoto Encyclopedia of Genes and Genomes) orthologs associated with the butyrate synthesis pathway and upstream pathways, including Gluconeogenesis, TCA cycle, and Fatty acid synthesis. Red color indicates significantly up-regulated enzyme commission (EC) numbers in the MR group, while green color represents significantly up-regulated EC numbers in the BM group. **F** Enrichment disparities in third-level metabolic pathways based on KEGG analysis in the goat colon. The red circle signifies metabolic pathways significantly enriched in the MR group, while the blue circle represents pathways significantly enriched in the BM group. Statistical significance was determined using the Dunn Test, with a significance level set at* P* < 0.001. **G** Correlations between the composition of gut microbiota at the species level and the concentrations of serum and liver parameters. Positive correlations are denoted in red, while negative correlations are represented in green

To further investigate the effects of milk replacer feeding treatment on gut microbial composition and function in goat kids and the correlation between the colon microbes with lipid metabolism. The variety of the bacteria in the colon was examined in the two groups. According to the Shannon and Chao index, the MR group generally had less alpha diversity at the species level than the BM group (*P* = 0.02, Additional file 1: Fig. S3A). Principal coordinate analysis (PCoA) at species level showed a clear separation of microbial community structure between the two groups (ANOSIM, *r* = 0.996, *P* = 0.001, Fig. 3A). We also analyzed variations in microbial communities between the two groups at the phylum level, and we found that the abundance of Firmicutes was significantly lower in the MR groups than in the BM group. In contrast, the abundance of Actinobacteria was significantly higher in the MR groups than in the BM group (Fig. 3B). At the genus level, the abundance of *Megamonas*, *Bifidobacterium*, *Prevotella*, and *Parabacteroides* were significantly increased in the MR group (*P* < 0.05, Additional file 1: Fig. S2B), however, the abundance of *Clostridium*, *Subdoligranulum*, *Faecalibacterium*, *Eubacterium* and *Lachnoclostridium* were significantly decreased in the MR group (*P* < 0.05, Additional file 1: Fig. S2B). Using LEfSe analysis to identify additional key signature species, it was discovered that MR goats had significantly higher levels of *Bacteroides plebeius* CAG 211, *Lactobacillus mucosae*, *Bacteroides coprocola* CAG 162, *Bifidobacterium longum*, *Prevotella* sp. 109, *Ruminococcus gnavus* CAG 126, and *Megamonas funiformis* CAG 377 (LDA > 3.5, Fig. 3C; Additional file 3: Table S6), and BM goats had significantly higher levels of *Bacteroides vulgatus*, *Subdoligranulum variabile*, *Faecalibacterium prausnitzii*, *Ruminococcaceae bacterium* AM2, *Flavonifractor plautii*, *Clostridia bacterium* UC5.1-1D1, *Eubacterium desmolans*, *Staphylococcus* sp. CAG 324, *Lactobacillus reuteri*, *Pseudoflavonifractor capillosus*, and *Butyricimonas virosa* (LDA > 3.5, Fig. 3C). Further analysis of the differences in colonic microbial interaction networks between the two groups revealed that the MR group formed an interaction network with core species including *Bacteroides plebeius*, *Escherichia coli*, *Bacteroides finegoldii*, *Bacteroides coprophilus*, *Parabacteroides distasonis*, *Olsenella *sp. DNF00959, *Ruminococcaceae bacterium *GD1, *Sutterella wadsworthensis*, and *Blautia* sp. KLE 1732 (Degree Centrality > 0.45; Additional file 1: Fig. S4), while the BM group formed an interaction network with core species including *Clostridia bacterium *UC5.1-1D1, *Eubacterium desmolans*, *[Ruminococcus] gnavus*, *Lactobacillus amylovorus*, *Alistipes *sp. HGB5, *Alistipes finegoldii*, *Firmicutes bacterium CAG:424*, *Clostridium* sp. ATCC BAA-442, and *Blautia *sp. KLE 1732 (Degree Centrality > 0.45; Additional file 1: Fig. S4). This further confirms the influence of milk replacer feeding on the core structure of colonic microbiota.

At the functional level, the microbiota in the MR group was mainly enriched in pathways related to ‘starch and sucrose metabolism’, ‘fructose and mannose metabolism’, ‘biosynthesis of amino acids’, ‘phenylpropanoid biosynthesis’, ‘oxidative phosphorylation’, and ‘beta-Lactam resistance’, while the microbiota in the BM group was mainly enriched in ‘ABC transporters’, ‘pyruvate metabolism’, ‘aminoacyl-tRNA biosynthesis’, and ‘amino sugar and nucleotide sugar metabolism’ (Fig. 3F). Further analysis of the differences in the abundance of CAZyme genes encoding carbohydrate enzymes in the colonic microbiota revealed that, compared to the BM group, the MR group showed significantly upregulated enzyme gene expression related to the synthesis of glucose from butyrate (Fig. 3D), as well as enzymes involved in the degradation of L-leucine, L-valine, and L-isoleucine, which are precursors for gluconeogenesis, TCA cycle, and gatty acid synthesis (Fig. 3E). This further confirms that disturbances in the core structure of colonic microbiota caused by milk replacer feeding may potentially affect the host’s absorption and utilization of energy and lipid nutrients.

The correlations between the level of serum triglyceride (TG) and cholesterol (TC) and liver triglyceride, cholesterol and free fatty acid (NEFA) and changes in microbial abundance were evaluated by Spearman’s correlation analysis. We found that liver cholesterol and liver free fatty acid concentrations positive correlated with *Clostridia bacterium *UC5.1-1D1, *Eubacterium desmolans*, *Flavonifractor plautii*, *Ruminococcaceae bacterium *AM2, *Faecalibacterium prausnitzii*, *Lactobacillus reuteri* and *Subdoligranulum variabile*, negatively correlated with *Bacteroides plebeius *CAG:211, *Bacteroides coprocola*, *Bacteroides plebeius*. Besides, *Bacteroides fragilis* abundance positively correlated with liver NEFA, *[Ruminococcus] gnavus* negatively correlated with liver cholesterol concentrations, and *Butyricimonas virosa* positively correlated with liver cholesterol concentrations (Fig. 3G). In conclusion, these results indicated that the changes in colonic microbial composition and function during milk replacer feeding may potentially impact the host’s lipid metabolism capabilities.

### Milk replacer feeding affect the colonic content lipid metabolism pathways in goat kids

**Fig. 4:**
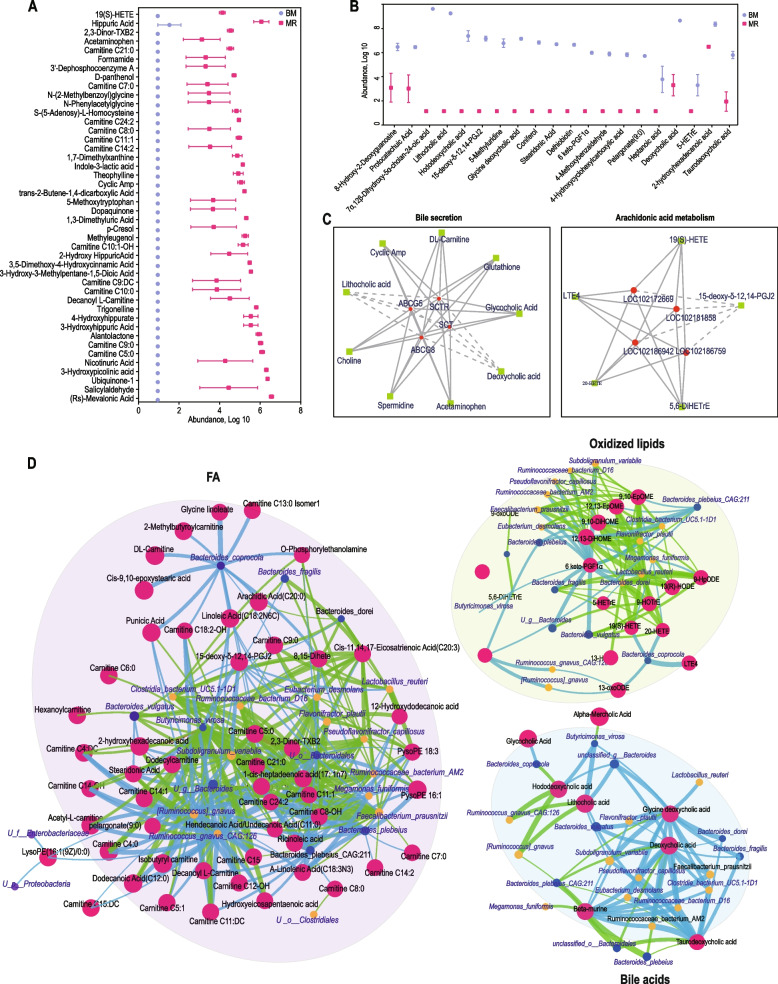
Impact of formula feeding on colonic bile acid profile in goat kids. **A **and** B** Relative abundance of significantly different metabolites in the colon of the BM (Breast Milk) and MR (Formula Feeding) groups. **C** Correlations between representative metabolites and genes were analyzed, and the outcomes of differential gene and differential metabolite correlations with Pearson’s correlation coefficients exceeding 0.80 and *P*-values below 0.05. **D** Correlations between the composition of gut microbiota at the species level and representative metabolites. Positive correlations are depicted in red, while negative correlations are illustrated in green

We analysed the effect of milk replacer feeding on differential metabolites in the colonic contents of goat kids using an LC–ESI–MS/MS system. A total of 272 metabolites were significantly altered in the MR group compared to the BM group, of which 56 metabolites were significantly downregulated and 216 metabolites were significantly upregulated (Additional file 4: Table S7). Interesting, 44 metabolites were undetectable in the BM group, while they exhibited a high abundance in the MR group, primarily including carnitine and fatty acid-like compounds (Rs-mevalonic acid, salicylaldehyde, 3-hydroxypicolinic acid, nicotinuric acid, and 3-hydroxyhippuric acid) (Fig. 4A; Additional file 4: Table S7). Furthermore, 15 metabolites were exclusively abundant in the BM group and were not detectable in the MR group, mainly belonging to the bile acid class (lithocholic acid, hododeoxycholic acid, glycine deoxycholic acid, and deoxycholic acid) (Fig. 4B). We further calculated Spearman’s rho coefficients between the DEG and colonic luminal metabolites, and coefficients greater than 0.8 with a *P*-value < 0.05 were considered significant. We found that the lipid metabolism genes *ABCG5*, *ABCG8*, *SCTR* and *SCT* were positively correlated with the metabolites choline, spermidine, acetaminophen, glycocholic acid, glutathione, DL-carnitine and cyclic amp involved in the bile acid secretion pathway and negatively correlated with deoxycholic acid and lithocholic acid. LOC102172669, LOC102186942, LOC102181858 and LOC102186759 genes were positively correlated with the metabolites LTE4, 19(S)-HETE, 5,6-DiHETrE and 20-HETE involved in the arachidonic acid metabolism pathway and negatively correlated with 15-deoxy-δ-12,14-PGJ2 (Fig. 4C).

To further analyze the correlation between differential metabolites and distinct colonic microbiota, Spearman’s coefficients between differential microbial and metabolites were then calculated, and coefficients greater than 0.8 with a *P*-value < 0.05 were considered significant. Interestingly, we found *Bacteroides coprocola* abundance positively correlated with carnitine C13:0 Isomer1, glycine linoleate, 2-methylbutyroylcarnitine, DL-carnitine, *cis*-9,10-epoxystearic acid, punicic acid, linoleic acid C18:2N6C, carnitine C18:2-OH and O-phosphorylethanolamine involved in the FA. *Ruminococcus gnavus *CAG:126 abundance positively correlated with carnitine. Among the metabolites of oxidized lipid, *Bacteroides plebeius *CAG:211 positively correlated with 9,10-EpOME, 12,13-EpOME, 9,10-DiHOME, 12,13-DiHOME and 6-keto-PGF1α. *Lactobacillus reuteri*, *Megamonas funiformis* and *Bacteroides dorei* negatively correlated with 9-HOTrE, 13(R)-HODE, 20-HETE and 9-HpODE. *Lactobacillus reuter* positively correlated with glycine deoxycholic acid. *Faecalibacterium prausnitzii*, *Pseudoflavonifractor capillosus*, *Ruminococcaceae bacterium *D16, *Eubacterium desmolans* and *Subdoligranulum variabile* positively correlated with β-murine, taurodeoxycholic acid, deoxycholic acid and glycine deoxycholic acid involved in the bile acids. *Ruminococcus gnavus *CAG:126 negatively correlated with hododeoxycholic acid and lithocholic acid (Fig. 4D). These findings indicated that colon microbiota reshaping induced by milk replacer feeding leads to a deficiency in colonic secretion of certain secondary bile acids.

### Milk replacer feeding affect the serum metabolism pathways in goat kids

**Fig. 5:**
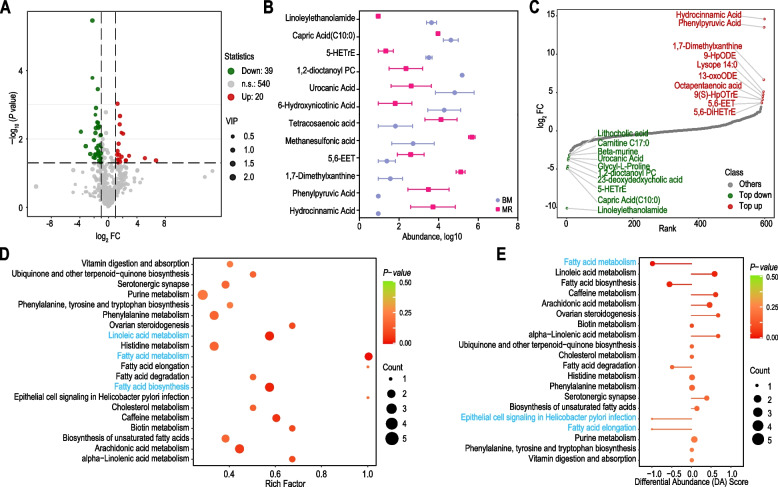
Influence of formula feeding on serum metabolites in goat kids. **A** Volcano plots displaying the results of metabolite analyses comparing the serum of the BM vs. MR group. Red indicates upregulated metabolites in the MR group, while green represents downregulated metabolites. **B** Relative abundance of significantly different metabolites in the colon of the BM and MR groups. **C** The top 10 differentially expressed serum metabolites in the BM vs. MR comparison. **D** Significant alterations in metabolic pathways in the MR group. **E** Analysis of Differential Abundance (DA) scores for KEGG pathways in the BM vs. MR comparison

Untargeted metabolome profiles were generated on goat serum samples using an LC–ESI–MS/MS system to assess the effect of milk replacer on differential metabolites in serum. We found 39 metabolites were significantly downregulated, and 20 metabolites were significantly upregulated in MR group (Fig. 5A; Additional file 5: Table S8). Among them, 1,7-dimethylxanthine, 9-HpODE, Lysope 14:0, 13-oxoODE, octapentaenoic acid, 9(S)-HpOTrE, 5,6-EET and 5,6-DiHETrE were the top 10 upregulated metabolites in the MR group. Lithocholic acid, carnitine C17:0, β-murine, urocanic acid, glycyl-L-proline, 1,2-dioctanoyl PC, 23-deoxydeoxycholic acid, 5-HETrE, capric acid (C10:0) and linoleylethanolamide were the top 10 downregulated metabolites in the MR group. Interestingly, we found that some metabolites were in high relative abundance in the BM group such as urocanic acid, 1,2-dioctanoyl PC, 5-HETrE, capric acid C10:0, linoleylethanolamide and 6-hydroxynicotinic acid, while phenylpyruvic acid, 1,7-Dimethylxanthine, 5,6-EET, methanesulfonic acid, tetracosaenoic acid and hydrocinnamic acid were in high abundance in the MR group (Fig. 5B and C; Additional file 5: Table S8).

The KEGG compound database generated the KEGG enrichment to describe the effect of milk replacer on these responsive metabolites and revealed that the ‘arachidonic acid metabolism’, ‘biosynthesis of unsaturated fatty acids’, ‘cholesterol metabolism’, ‘fatty acid biosynthesis’, ‘fatty acid degradation’, ‘fatty acid elongation’, ‘fatty acid metabolism’, ‘linoleic acid metabolism’, and ‘alpha-linolenic acid metabolism’ were the most significantly affected pathways (Fig. 5D). The metabolic pathways significantly downregulated were ‘fatty acid elongation’, ‘fatty acid metabolism’ and ‘epithelial cell signaling in helicobacter pylori infection’ in the MR group (Fig. 5E). This further corroborates that milk replacer feeding significantly disrupts the concentrations of metabolites involved in lipid metabolism in the serum, thereby impacting the host’s lipid metabolism capacity.

### Formula feeding affect the lipid metabolism profile is transferable by IMT

**Fig. 6:**
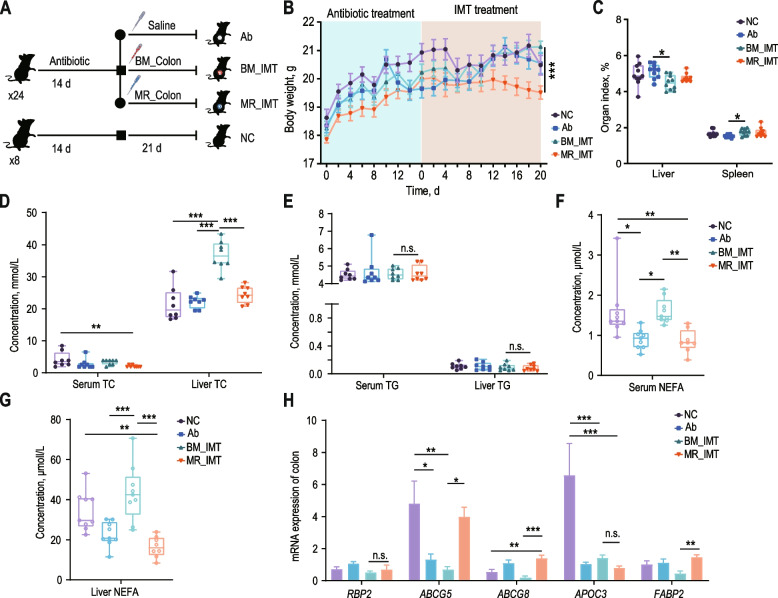
Transference of altered lipid metabolism profile by formula feeding through intestinal microbiota transplantation. **A** Schematic representation of the experimental design. **B** Body weight of SPF C57/6 J mice. Data differences were assessed using one-way analysis of variance (ANOVA) with Tukey’s test. **C** Organ indexes of SPF C57/6 J mice. **D** Concentration of total cholesterol (TC) in the serum and liver of SPF C57/6 J mice. **E** Concentration of triglycerides (TG) in the serum and liver of SPF C57/6 J mice. **F** and **G** Concentration of non-esterified fatty acids (NEFA) in the serum and liver of SPF C57/6 J mice. **H** Expression of mRNA related to lipid metabolism in the colon of the mouse model. Data differences were analyzed using one-way ANOVA with Tukey’s test. Significance levels are indicated as follows: n.s. (not significant) for *P* > 0.05, * for *P* < 0.05, ** for *P* < 0.01, and *** for* P* < 0.001

To validate the causal relationship between milk replacer feeding-induced colonic microbiota disruption and host lipid metabolism, we transferred the colon microbiota from BM and MR group to bacterial-restricted SPF C57/6 J mice (Fig. 6A). Compared with BM_IMT mice, the weight of MR_IMT mice decreased from day 12 of transplantation, and the weight difference between the two groups was significant at d 20 of transplantation (Fig. 6B). The organ indexes of liver and spleen of BM_IMT and MR_IMT mice showed no significant difference (Fig. 6C). To clarify the causal relationship between hindgut microbes and lipid metabolism in goat, we evaluated the effects of microbiota on lipid metabolism ability of mice. The levels of TC, TG and NEFA indictor were measured in serum and liver samples and revealed that MR_IMT group significantly decreased TC concentrations in liver and NEFA concentrations in serum and liver compared with the BM_IMT group but no significant effect on TG concentration in serum and liver (Fig. 6D–G). The mRNA expression level of genes associated with lipid metabolism in the NC, Ab, BM_IMT and MR_IMT treatment groups was further investigated. There was a significant increase in the mRNA expression of *ABCG5*, *ABCG8* and *FABP2* in the MR_IMT group, while the mRNA expression of *RBP2* and *APOC3* showed no significant difference (Fig. 6H).

We conducted 16S rRNA gene sequencing to examine the colon bacteria composition to confirm that fecal transplantation modulates the gut microbiota. Our results indicated that α-diversity (via Chao and Pd Index) and β-diversity were not significantly different in the BM_IMT and MR_IMT group (Additional file 1: Fig. S5A and B). At the genus level, the abundance of *Bacteroides*, *Colidextribacter*, *Parabacteroides* and *Escherichia-Shigella* were enriched in the MR_IMT group, while *u_f_Eggerthellaceae* and *Holdemania* were enriched in the BM_IMT group (Additional file 1: Fig. S5C). The ASV-level analysis showed that the relative abundance of ASV131 (*Bacteroidaceae*), ASV266 (*Lachnospiraceae*) were detected in the BM_IMT group. Moreover, the relative abundance of ASV398 (*Muribaculaceae*), ASV94 (*Muribaculaceae*), ASV6 (*Muribaculaceae*), ASV233 (*Clostridia_UCG-014*), ASV472 (*Clostridia_vadinBB60*), ASV993 (*Sutterellaceae*), ASV383 (*Sutterellaceae*) were not identified in the BM_IMT group (Additional file 1: Fig. S5D). These results causally confirm that milk replacer feeding-induced colonic microbiota disruption is associated with a phenomenon that can affect the host’s lipid metabolism capacity, with potential cross-species implications.

## Discussions

**Fig. 7:**
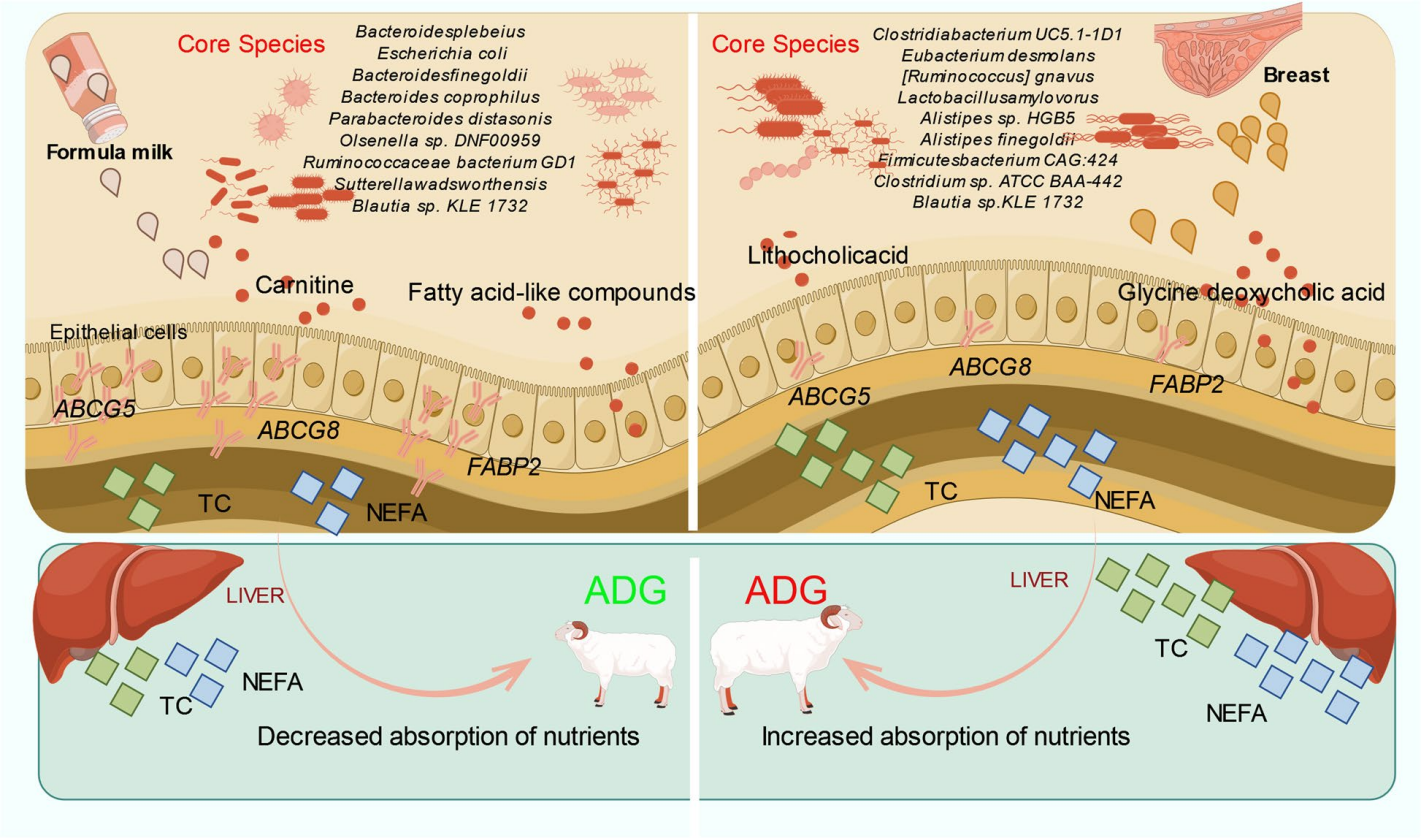
Process of formula feeding-mediated disruption of goat kid lipid metabolism through modulation of gut microbiota and bile acid secretion. Figdraw software was used to create this illustration

The purpose of this study was to understand how milk replacer feeding alters the colon microbiota in goat kids and affects their lipid metabolism, with a focus on the potential modulation of the host’s lipid metabolism pathways. Our results demonstrated that milk replacer feeding significantly reduced daily weight gain and decreased the concentration of cholesterol and free fatty acids in the serum and liver of goat kids. This was evident from multiple omics analyses, including metagenomic sequencing, metabolic profiling, RNA-seq analysis, and colonic digesta transplantation. These findings suggest that milk replacer feeding affects the microbial composition and metabolite characteristics, directly influencing the differential expression of colon lipid metabolism genes, thereby affecting the lipid metabolism pathway (Fig. 7).

A key observation was that milk replacer feeding led to significant changes in the gut microbiota composition. Specifically, we noted a disruption in the abundance of *Bacteroides* spp., which are known to play a crucial role in cholesterol metabolism [30–32]. This disruption likely contributed to the substantial changes in bile acid levels observed in our study. Correlation analyses between microbiota and bile acid metabolites supported this viewpoint, indicating that the presence of *Bacteroides* spp. could be beneficial in mitigating the negative effects on lipid metabolism seen with milk replacer feeding. Furthermore, our study found that the milk replacer feeding regimen did not alter the concentrations of serum and hepatic triglycerides but resulted in a reduction in cholesterol levels. This phenomenon may be attributed to the lower cholesterol content in milk replacer compared to breast milk, which may downregulate the generation of hepatic hydroxymethylglutaryl coenzyme A reductase, thereby reducing endogenous cholesterol synthesis [33–35].

The analysis of bile acids revealed the absence of certain secondary bile acids in the colon and serum of goats fed with milk replacer. This absence inhibited fat emulsification, reduced pancreatic lipolysis, and hindered the absorption of lipid substances in the intestine, ultimately impacting growth and development [36]. The lack of these bile acids likely led to an impaired lipid metabolic pathway, as indicated by the downregulation of genes associated with lipid metabolism in the colonic epithelium. Additionally, the study confirmed that milk replacer feeding induced an upregulation of *ABCG5* and *ABCG8* expression in the colonic epithelium. These genes are involved in cholesterol reverse transport, small intestine absorption, and bile acid secretion, interacting with dietary components to regulate blood cholesterol levels and maintain cholesterol metabolic homeostasis [37–39].

Overall, our findings highlight the impact of milk replacer feeding on the core composition of colonic microbiota and its subsequent effect on the expression profile of colonic lipid metabolism genes. These alterations influence the host’s lipid metabolic program and affect normal goat growth and development. Further investigation is needed to elucidate the mechanisms involving specific functional microbiota and the regulation of colonic epithelial lipid metabolism gene expression by key secondary bile acids.

## Conclusions

Our study demonstrates that milk replacer feeding in goats profoundly influences colonic lipid metabolism and microbial ecology, which are crucial for overall metabolic health and development. We observed that milk replacer feeding reduced serum and liver concentrations of cholesterol and free fatty acids, thereby downregulating colonic lipid absorption and cholesterol transport pathways. These alterations are particularly impactful during the critical growth phase of kids, affecting their normal development. Furthermore, milk replacer feeding induced significant changes in the core microbial species composition in the colon, notably leading to the absence of secondary bile acids. Utilizing a germ-free mouse model validated a causal relationship between disrupted colonic microbiota induced by milk replacer feeding and key metabolic parameters such as daily weight gain, circulating cholesterol, free fatty acid levels, and the expression of colonic lipid metabolism genes. In summary, our findings underscore the detrimental effects of milk replacer feeding on colonic lipid metabolism through microbiota disruption in goats. This study provides novel insights into the mechanisms through which milk replacer feeding impacts colon metabolism and physiology in ruminants. These insights are pivotal for developing new milk replacer formulations enriched with active probiotics and prebiotics. Such formulations could potentially mitigate the adverse effects observed here by targeting specific bioactive substances and fostering beneficial bacterial strains.

## Supplementary Information

**Additional file 1: Table S1**. Ingredients of the experimental diets of mother goats. **Table S2**. Ingredients of the experimental diets of goat kids (DM basis). **Table S3**. Ingredients of the experimental diets of mice (DM basis). **Table S4**. qPCR primers used for gene expression analysis. **Fig. S1**. Serum Stress markers, inflammatory cytokines and immune cytokines concentration in goats under different feeding conditions. **Fig. S2**. Transcriptional profiling of colonic epithelium in response to formula feeding. **A** Gene Ontology (GO) enrichment analysis of genes in the differentially expressed gene set. **B** Network representation of GO enrichment analysis. **Fig. S3**. Microbial diversity analysis and relative abundance of bacterial genera in Breast Milk (BM) and Formula Feeding (MR) Groups. **A** Alpha diversity analysis based on the species level. **B** Relative abundance of bacterial genera in the BM (Breast Milk) and MR (Formula Feeding) groups. Red asterisks indicate significant differences in bacterial abundance between the two groups. **Fig. S4**. Microbial interaction network in the goat colon under different feeding additives. **Fig. S5**. Impact of gut microbiota transplantation on mouse gut microbiome composition. **A **Alpha diversity analysis at the ASV level. **B **Principal Coordinate Analysis (PCoA) plot based on ASV level, with box plots illustrating Bray–Curtis distances associated with groupings, assessed using the Wilcoxon rank-sum test. Analysis of similarity (ANOSIM) was employed to evaluate the dissimilarity of Bray–Curtis distances. **C **Relative abundance of bacterial genera in the four groups. Red asterisks indicate significant differences in bacterial abundance between the MR_IMT and MR_IMT groups. **D **Selection of microbiota categories with significantly different abundance in the MR_IMT and MR_IMT groups.

**Additional file 2:** **Tables S5**. The information of DEGs between BM and MR group in goats.

**Additional file 3:** **Table S6**. Differential microbiota between BM and MR goat kids in species level.

**Additional file 4:** **Table S7**. Unique list of 272 colon metabolites altered comparing BM and MR goat kids.

**Additional file 5: Table S8**. Unique list of 138 serum metabolites altered comparing BM and MR goat kids.

## Data Availability

The metagenomic sequencing and RNA-seq data are available from the national center for biotechnology information (NCBI) under accessions PRJNA1029896 (https://dataview.ncbi.nlm.nih.gov/object/PRJNA1029896?reviewer=f8oisljdbf1sdgvmu69er8df15) and PRJNA1030534 (https://dataview.ncbi.nlm.nih.gov/object/PRJNA1030534?reviewer=l6t3793h9q753mangoer3cl3rm), respectively.

## Abbreviations

ABC: ATP-binding cassette
BM: Breast milk
Cor: Cortisol
Cort: Corticosterone
DAO: Diamine oxidase
Glu: Glucose
HIF-α: Hypoxia-inducible factor
IgA: Immunoglobulin A
IgG: Immunoglobulin G
IgM: Immunoglobulin M
IL-6: Interleukin-6
IL-10: Interleukin-10
IL-1β: Interleukin-1β
IMT: Intestinal microbiota transplantation
LDA: Linear discriminant analysis
LEfSe: Linear discriminant analysis effect size
MR: Milk replacers
NEFA: Non-esterified fatty acid
PCoA: Principal coordinate analysis
PBS: Phosphate-buffered saline
RIN: RNA integrity numbers
TC: Total cholesterol
TG: Triglycerides
TNF-α: Tumor necrosis factor-α
