# Supplementary material for: Early-life milk replacer feeding mediates lipid metabolism disorders induced by colonic microbiota and bile acid profiles to reduce body weight in goat model
Source: J Anim Sci Biotechnol. 2024 Sep 4;15:118. doi: 10.1186/s40104-024-01072-x (PMC11373095; doi:10.1186/s40104-024-01072-x)
Supplement: Supplementary file 1 — Additional file 1: Table S1. Ingredients of the experimental diets of mother goats. Table S2. Ingredients of the experimental diets of goat kids (DM basis). Table S3. Ingredients of the experimental diets of mice (DM basis). Table S4. qPCR primers used for gene expression analysis. Fig. S1. Serum Stress markers, inflammatory cytokines and immune cytokines concentration in goats under different feeding conditions. Fig. S2. Transcriptional profiling of colonic epithelium in response to formula feeding. A Gene Ontology (GO) enrichment analysis of genes in the differentially expressed gene set. B Network representation of GO enrichment analysis. Fig. S3. Microbial diversity analysis and relative abundance of bacterial genera in Breast Milk (BM) and Formula Feeding (MR) Groups. A Alpha diversity analysis based on the species level. B Relative abundance of bacterial genera in the BM (Breast Milk) and MR (Formula Feeding) groups. Red asterisks indicate significant differences in bacterial abundance between the two groups. Fig. S4. Microbial interaction network in the goat colon under different feeding additives. Fig. S5. Impact of gut microbiota transplantation on mouse gut microbiome composition. A Alpha diversity analysis at the ASV level. B Principal Coordinate Analysis (PCoA) plot based on ASV level, with box plots illustrating Bray–Curtis distances associated with groupings, assessed using the Wilcoxon rank-sum test. Analysis of similarity (ANOSIM) was employed to evaluate the dissimilarity of Bray–Curtis distances. C Relative abundance of bacterial genera in the four groups. Red asterisks indicate significant differences in bacterial abundance between the MR_IMT and MR_IMT groups. D Selection of microbiota categories with significantly different abundance in the MR_IMT and MR_IMT groups. [file 40104_2024_1072_MOESM1_ESM.docx]

Supplementary Materials for

**Early-life milk replacer feeding mediate lipid metabolism disorders induced by colonic microbiota and bile acid profiles to reduce body weight in goat model**

Ke Zhang *et al.*

Corresponding authors: Yulin Chen, [chenyulin@nwafu.edu.cn](mailto:chenyulin@nwafu.edu.cn);

**The PDF file includes:**

Table S1 to S4

Fig. S1 to S5

| **Table S1. Ingredients of the experimental diets of mother goats.** | |
| --- | --- |
| **Item** | **Content (% of DM)** |
| **Nutritional ingredient (% of DM）** |  |
| Corn | 47.08 |
| Soybean meal | 17.80 |
| Wheat | 8.00 |
| Wheat bran | 5.00 |
| Alfalfa meal | 5.00 |
| Highland barley | 10.00 |
| Methionine | 0.02 |
| Zeolite powder | 0.50 |
| Vitamin | 0.10 |
| Yeast extract | 3.00 |
| Premix | 1.00 |
| Water-soluble chloride | 1.00 |
| Calcium + Phosphorus | 1.50 |
| Total | 100.00 |
| **Nutritional level (% of DM)** |  |
| Organic matter | 80.97 |
| Crude protein | 14.97 |
| Crude fat | 2.65 |
| Crude fiber | 7.21 |
| Nitrogen-free extract | 51.91 |
| Ash | 2.73 |
| Neutral detergent fiber | 14.05 |
| Acid detergent fiber | 6.52 |

**Table S2 Ingredients of the experimental diets of goat kids (%, DM basis).**

| **Animals** | **Item** | **Content** |
| --- | --- | --- |
| **Goat** | Water（%） | ≤6.00 |
|  | Crude protein（%） | ≥27.00 |
|  | Crude fat（%） | ≥18.00 |
|  | Ash（%） | ≤10.00 |
|  | Crude fiber（%） | ≤3.00 |
|  | Calcium（%） | 0.60 |
|  | Phosphorus（%） | 0.50 |
|  | Sodium chloride（%） | 0.10 |
|  | Lysine（%） | ≥2.20 |
|  | Methionine（%） | ≥1.00 |
|  | Threonine（%） | ≥1.00 |
|  | Vitamin E（IU） | ≥80.00 |

**Table S3 Ingredients of the experimental diets of mice (%, DM basis).**

| **Animals** | **Item** | **Content** |
| --- | --- | --- |
| **Mice** | Dry matter（%） | 85.00 |
|  | Crude protein（g/kg） | 200.00 |
|  | Crude fat（g/kg） | 40.00 |
|  | Crude fiber（g/kg） | 50.00 |
|  | Ash（g/kg） | 80.00 |
|  | Calcium（g/kg） | 15.00 |
|  | Lysine（g/kg） | 9.00 |
|  | Lysine（g/kg） | 13.20 |
|  | Methionine + Cysteine（g/kg） | 7.80 |

**Table S4 qPCR primers used for gene expression analysis.**

| Species | Gene symbol | Forward（5’-3’） | Reverse（5’-3’） | Product length |
| --- | --- | --- | --- | --- |
| Mice | *FABP2* | TGGAAAGGAGCTGATTGCTGT | TGGAGACCAGTGCTGATAGGA | 214 |
|  | *RBP2* | CTACGACCTGGATTTCACCGTC | ACTGCTTCCAGCCACGGTTCTC | 149 |
|  | *ABCG8* | GGTCCTTCTGATGACATCTGGC | CGTCTGTCGATGCTGGTCAAGT | 144 |
|  | *ABCG5* | TGCCATCCTGACTTACGGAGAG | CTGCTTTGGGTGTCCACTGATG | 147 |
|  | *APOC3* | AAGACGGTCCAGGATGCGCTAA | GTTGGTCCTCAGGGTTAGAATCC | 157 |
|  | β-actin | AGGGAAATCGTGCGTGACAT | GGAAAAGAGCCTCAGGGCAT | 172 |


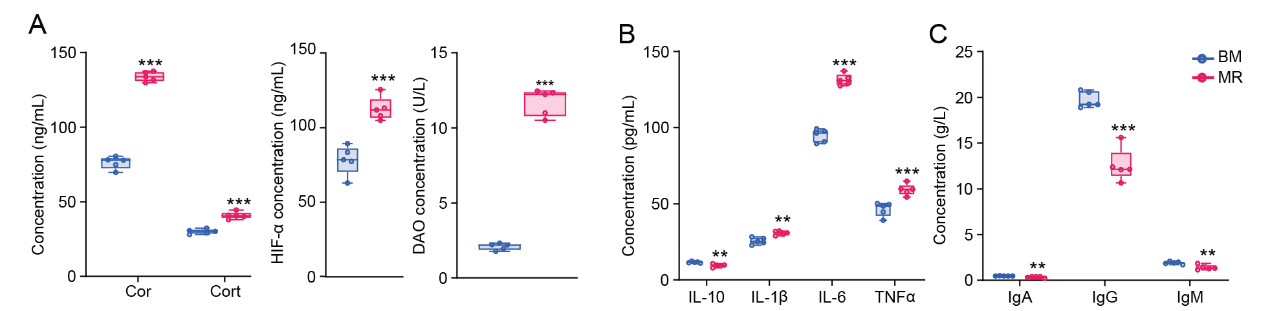
**Fig. S1** Serum Stress markers, inflammatory cytokines and immune cytokines concentration in goats under different feeding conditions.

**
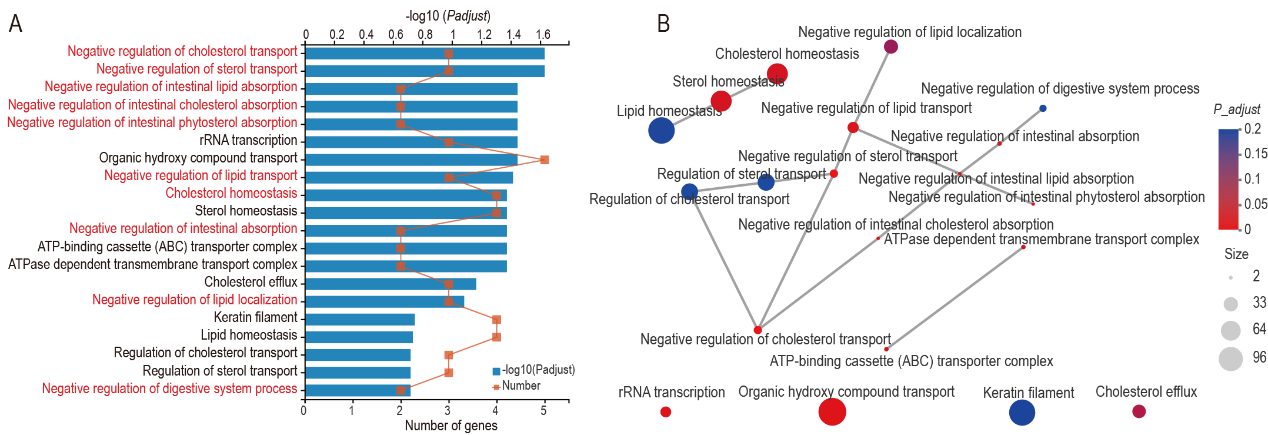
**

**Fig. S2. Transcriptional profiling of colonic epithelium in response to formula feeding.** (A) Gene Ontology (GO) Enrichment Analysis of genes in the differentially expressed gene set. (B) Network representation of GO enrichment analysis.

**
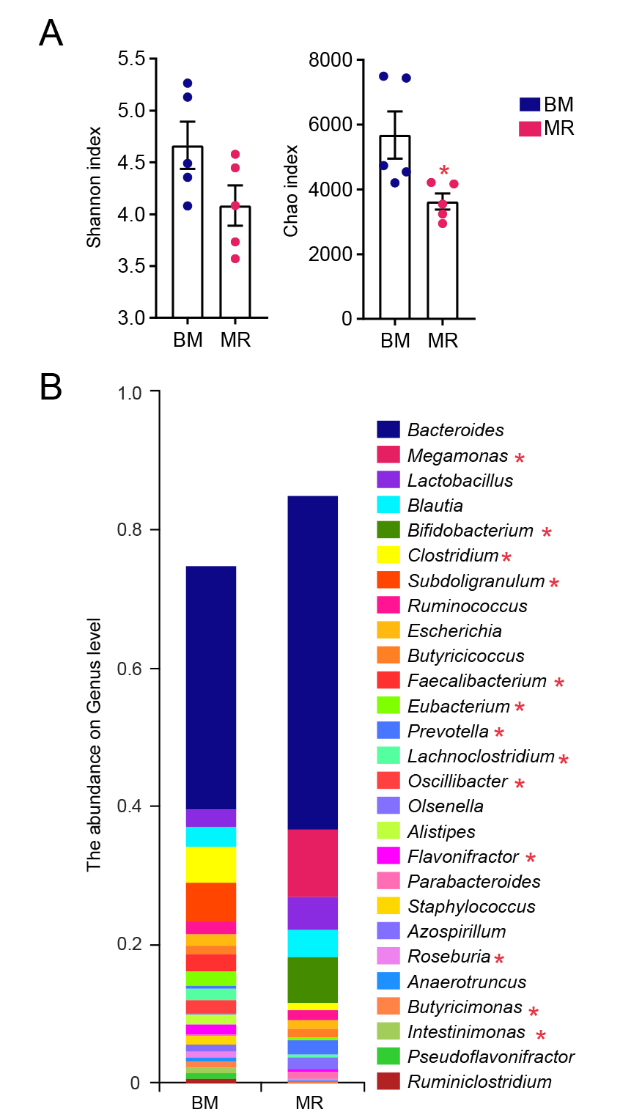
**

**Fig. S3. Microbial diversity analysis and relative abundance of bacterial genera in breast milk (BM) and formula feeding (MR) groups.** (A) Alpha diversity analysis based on the species level. (B) Relative abundance of bacterial genera in the BM (Breast Milk) and MR (Formula Feeding) groups. Red asterisks indicate significant differences in bacterial abundance between the two groups.


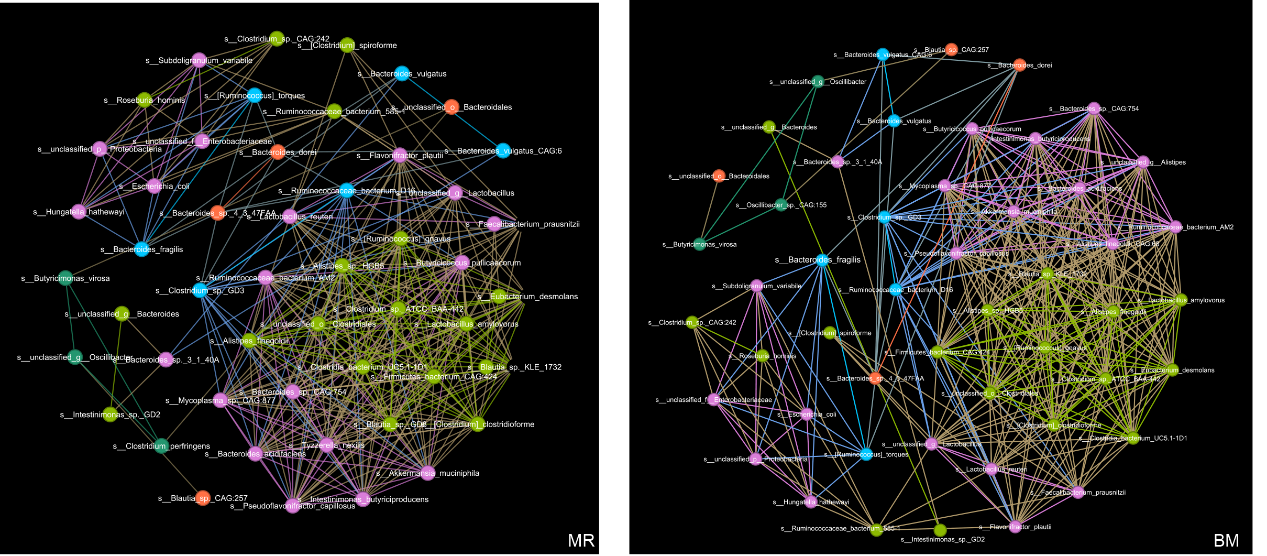


**Fig. S4. Microbial interaction network in the goat colon under different feeding additives.**


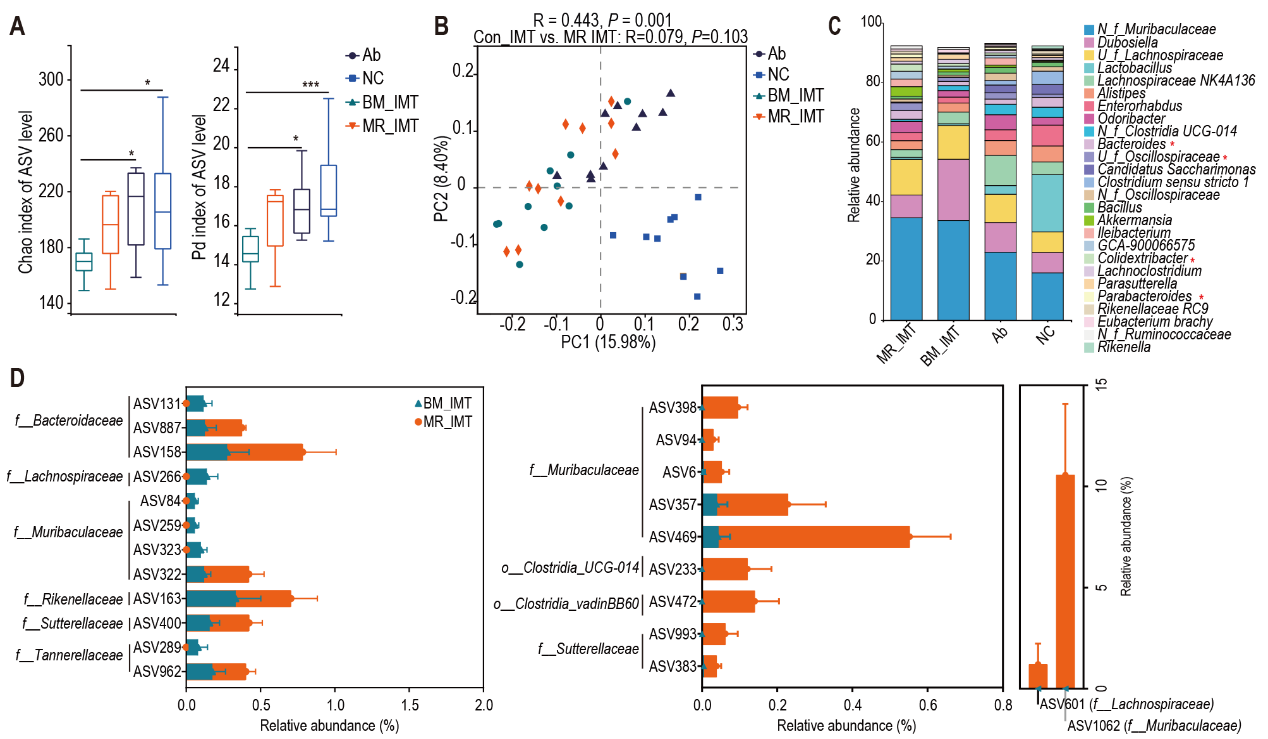


**Fig. S5. Impact of gut microbiota transplantation on mouse gut microbiome composition.** (A) Alpha diversity analysis at the ASV level. (B) Principal Coordinate Analysis (PCoA) plot based on ASV level, with box plots illustrating Bray–Curtis distances associated with groupings, assessed using the Wilcoxon rank-sum test. Analysis of similarity (ANOSIM) was employed to evaluate the dissimilarity of Bray–Curtis distances. (C) Relative abundance of bacterial genera in the four groups. Red asterisks indicate significant differences in bacterial abundance between the MR_IMT and MR_IMT groups. (D) Selection of microbiota categories with significantly different abundance in the MR_IMT and MR_IMT groups.
